# Vaccine Therapy for the Management of Penile Cancer: Evidence, Opportunities and Challenges

**DOI:** 10.3390/vaccines14070597

**Published:** 2026-07-06

**Authors:** Firas Hatoum, Ricardo Nehme, Adnan Fazili, Justin Miller, Jeffrey S. Johnson, Casey Le, Philippe E. Spiess, Jad Chahoud

**Affiliations:** 1 Department of Genitourinary Oncology and Tumor Biology, H. Lee Moffitt Cancer Center and Research Institute, Tampa, FL 33612, USA; firas.hatoum@moffitt.org (F.H.); adnan.fazili@moffitt.org (A.F.); justin.w.miller@moffitt.org (J.M.); casey.le@moffitt.org (C.L.); 2 Department of Medicine, Mayo Clinic, Jacksonville, FL 32224, USA; nehmefahmy.ricardo@mayo.edu; 3 Department of Genitourinary Oncology, Orlando Health Cancer Institute, Orlando, FL 32806, USA

**Keywords:** penile squamous cell carcinoma, cancer vaccines, human papilloma virus, neoantigens

## Abstract

Penile squamous cell carcinoma (PSCC) is a rare malignancy with limited therapeutic options in advanced and recurrent diseases. Advanced PSCC is typically managed with multimodal therapy, including neoadjuvant chemotherapy or chemoradiation followed by surgery; however, durable responses remain uncommon, and outcomes after recurrence are poor. Cancer vaccines represent a promising immunotherapeutic strategy, as these treatments induce tumor-specific immunity and heightened immune surveillance against penile cancer cells. While therapeutic cancer vaccines have not yet demonstrated consistent clinical efficacy as monotherapy in PSCC, their integration with complementary immune-modulating approaches, particularly immune checkpoint blockade, represents a rational strategy to enhance antitumor immunity. This review summarizes the rationale for vaccine development in PSCC, with emphasis on HPV-derived antigens, neoantigens, and emerging tumor-associated targets. We examine major vaccine platforms, including viral-vector, peptide-based, nucleic acid, and dendritic cell-based approaches. We also discuss how spatial transcriptomics, single-cell RNA sequencing, artificial intelligence-assisted antigen prediction, and nanotechnology-enhanced delivery systems may support future personalized vaccine development. Overall, therapeutic vaccines remain investigational in PSCC but may become relevant within biomarker-driven, combination-based immunotherapy strategies.

## 1. Introduction

**Figure 1:**
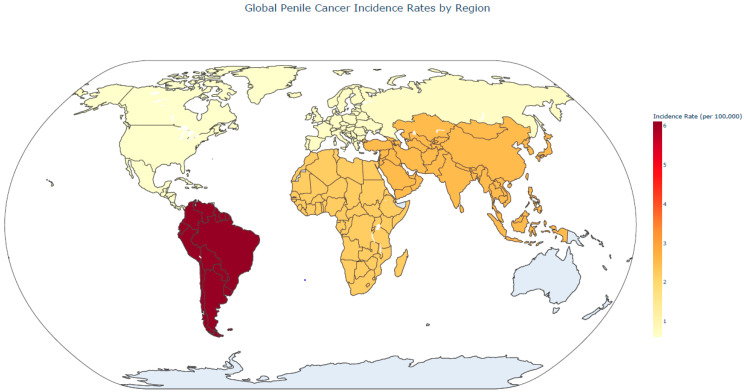
Penile cancer incidence rates by region (estimated for 2025). This figure presents the different incidence rates of penile cancer by region. Higher incidence rates were seen in South America, Southern Africa, and Southern Asia, where the disease may contribute up to 10% of the total malignancies [1,4,5].

Penile squamous cell carcinoma (PSCC) is a rare cancer with variable outcomes depending on the geographical distribution [1]. In the United States, approximately 2190 new cases of penile cancer were estimated in 2025, with squamous cell carcinoma accounting for most of them [1,2]. Globally, the age-standardized incidence of penile cancer has been estimated at approximately 0.8 per 100,000 men, with higher rates reported in parts of South America, sub-Saharan Africa, and South/Southeast Asia (Figure 1) [2].

PSCC primarily affects men over 50 years of age, with a mean age at diagnosis of 67 years [2,3,4,5]. Established risk factors include phimosis, smoking, HIV infection, and limited access to preventive healthcare. Human papillomavirus (HPV) infection, particularly high-risk serotypes 16 and 18, is implicated in approximately 30–50% of cases and plays a central oncogenic role through E6- and E7-mediated inactivation of p53 and Rb [6].

HPV contributes substantially to the global cancer burden and is etiologically linked to several anogenital and oropharyngeal malignancies. In penile cancer, systematic reviews have reported HPV DNA in approximately half of invasive PSCC cases and in a larger proportion of penile intraepithelial neoplasia cases [6,7,8].

While therapeutic approaches targeted towards the E6 and E7 proteins have been promising in other HPV-related malignancies (i.e., anal and cervical cancers), their extension to penile cancer is hampered by a lack of disease-specific data and clinical trials [9,10]. Moreover, in HPV-negative penile cancer, there is a need for additional investigation to elucidate the tumor immunopeptidome underlying such cancers and identify the complete set of potential tumor-associated antigens [11].

Therefore, while vaccine-based therapy is an inspiring new frontier in penile cancer therapy, particularly in the earlier cancer stages when the immune microenvironment remains potentially modulable, further research is immensely needed. This includes thorough genomic and immunopeptidomic profiling to identify actionable neoantigens [10], further characterization of immune evasion mechanisms [11], and rational combinatorial design of immunotherapy that may include vaccines, checkpoint inhibition, and immune reprogramming strategies [11]. A major challenge in vaccine development for PSCC is the rarity of the disease, which has limited the conduct of prospective vaccine-specific clinical trials. Consequently, much of the current understanding of therapeutic vaccination in PSCC is derived from translational studies and clinical experience in other HPV-associated malignancies, including cervical, anal, vulvar, and head and neck squamous cell carcinomas. Throughout this review, evidence originating from these malignancies is discussed within the context of its potential relevance to PSCC while recognizing the limitations of cross-disease extrapolation.

In this review, we summarize recent advances in the development and clinical implementation of vaccines for penile cancer. We discuss emerging strategies to induce antitumor immunity and the molecular mechanisms that support these approaches.

## 2. HPV-Associated and Tumor-Derived Vaccine Targets in PSCC

### 2.1. HPV-Derived Antigens

**Figure 2:**
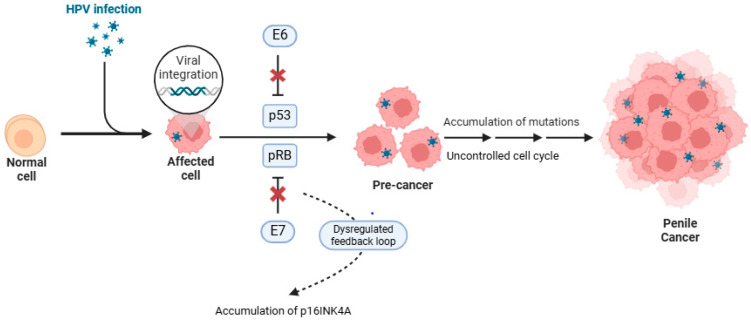
Mechanisms of HPV-mediated carcinogenesis in PSCC. High-risk HPV infection results in persistent expression of the viral oncoproteins E6 and E7 following viral genome integration. E6 promotes degradation of p53, impairing DNA damage responses and apoptosis, while E7 inactivates the retinoblastoma (Rb) pathway, resulting in dysregulated cell-cycle progression. Loss of Rb signaling leads to overexpression of p16INK4a, which serves as a surrogate marker of HPV-driven disease. The cumulative effects of these molecular alterations contribute to genomic instability and malignant transformation. E6 and E7 are attractive therapeutic vaccine targets because they are viral, tumor-associated, and required for maintenance of HPV-driven malignant phenotypes. In HPV-positive tumors, antigen presentation through MHC molecules can support activation of HPV-specific CD8+ cytotoxic T-cells and CD4+ helper T-cells. However, HPV-associated tumors may evade immune surveillance through impaired antigen presentation, altered interferon signaling, suppressive immune-cell recruitment, and upregulation of immune checkpoint pathways. These mechanisms provide a rationale for combining HPV-directed vaccines with immune checkpoint blockade or other immune-modulating approaches [15].

Integration of high-risk HPV DNA into the host genome can lead to persistent expression of the viral oncogenes E6 and E7. E6 promotes degradation of p53, impairing DNA damage responses and apoptosis, while E7 inactivates Rb, disrupting cell-cycle control and promoting uncontrolled proliferation (Figure 2) [12,13,14]. E7-mediated Rb pathway disruption also contributes to p16INK4a overexpression, which is commonly used as a surrogate marker of HPV-driven disease, although it is not itself the oncogenic driver.

Multiple studies in HPV-associated malignancies have identified immunogenic E6 and E7 epitopes capable of inducing HPV-specific T-cell responses [16]. HLA-restricted peptide studies have demonstrated that optimized or agonist epitopes can improve MHC binding, enhance IFN-γ secretion, and promote cytotoxic T-cell recognition of HPV-positive tumor cells. These findings have supported the development of peptide-based vaccines, DNA vaccines, RNA vaccines, viral-vector vaccines, and dendritic cell-based vaccines incorporating E6/E7 antigens. A key limitation of epitope-based vaccination is HLA restriction. Because antigen presentation depends on the patient’s HLA genotype, vaccine efficacy may vary across individuals. Multi-epitope and synthetic long-peptide strategies may broaden immune coverage by incorporating both CD8+ and CD4+ T-cell epitopes and reducing dependence on a single HLA allele.

### 2.2. Neoantigens and Oncofetal Antigens

Neoantigens, derived from somatic mutations, rank among the most distinct immunogenic molecules [17]. Neoantigen-directed vaccines have demonstrated immunogenicity and early clinical activity in selected malignancies such as melanoma, non-small cell lung cancer, and glioblastoma, particularly in tumors with higher mutational burden or microsatellite instability [18,19,20,21].

In PSCC, neoantigen-based vaccination remains highly investigational. Although genomic studies have identified recurrent molecular alterations and potential tumor-specific mutations in PSCC, no neoantigen has yet been clinically validated as a therapeutic vaccine target in this disease [20,22]. Moreover, the rarity of PSCC and the limited availability of comprehensive immunopeptidomic datasets have hindered systematic neoantigen discovery and validation efforts. Compared with highly mutated tumors such as melanoma and certain subsets of non-small cell lung cancer, PSCC may possess a more restricted neoantigen repertoire, potentially limiting the number of actionable targets available for personalized vaccine development [18,19,20,21,22]. Nevertheless, selected patients with higher tumor mutational burden, DNA repair alterations, or other genomic features associated with increased antigenicity may represent suitable candidates for individualized neoantigen-based approaches [22].

Other tumor-associated antigens, including cancer-testis antigens, differentiation antigens, and developmental or oncofetal antigens, may also be explored as potential vaccine targets. However, the relevance of oncofetal antigens in PSCC remains poorly characterized. Oncofetal antigens are developmental proteins that are normally expressed during embryogenesis but become largely silenced in adult tissues before being aberrantly re-expressed during malignant transformation [17]. While several oncofetal antigens have been investigated as immunotherapeutic targets in other solid tumors, there is currently no robust evidence supporting any specific oncofetal antigen as a validated vaccine target in PSCC [17]. Furthermore, antigen expression alone does not necessarily indicate clinical utility, as candidate antigens must also be processed and presented through major histocompatibility complex molecules and be capable of eliciting effective antitumor immune responses [22,23]. Consequently, oncofetal antigens should currently be regarded as exploratory targets in PSCC, requiring further validation through integrated genomic, proteomic, immunopeptidomic, and functional immune analyses before clinical application can be considered [10,17,22].

Future antigen discovery in PSCC should integrate genomic sequencing, transcriptomic profiling, proteomics, immunopeptidomics, and spatial immune profiling. This approach may help distinguish antigens that are merely expressed from those that are processed, presented, and spatially accessible to immune effector cells.

Emerging evidence suggests that non-canonical or cryptic antigens may further expand the pool of vaccine targets available for cancer immunotherapy. These antigens arise from alternative open reading frames, aberrant RNA splicing, intronic retention, untranslated regions, endogenous retroelements, and other non-conventional translation events that are not represented in the normal proteome. Because central immune tolerance is less likely to develop against these peptides, cryptic antigens may exhibit strong immunogenic potential and serve as attractive targets for therapeutic vaccination. Recent immunopeptidomic studies across multiple solid tumors have demonstrated that non-canonical peptides can be presented by major histocompatibility complex molecules and recognized by tumor-reactive T-cells. Although their role in PSCC remains unexplored, integration of immunopeptidomics, ribosome profiling, and advanced computational antigen discovery platforms may facilitate identification of cryptic antigens as future targets for personalized vaccine development [23].

## 3. Therapeutic Vaccine Development

Therapeutic vaccine development in PSCC remains in early stages, with most strategies extrapolated from other HPV-driven malignancies. Current approaches can be broadly categorized into vector-based, peptide-based, nucleic acid, and dendritic cell-based platforms, each with distinct advantages and limitations. Because prospective vaccine studies in PSCC remain extremely limited, much of the current rationale for therapeutic vaccination is derived from preclinical models and clinical experience in other HPV-associated malignancies, including cervical, anal, and head and neck cancers. Consequently, the approaches discussed below should be viewed as translational hypotheses and investigational strategies rather than established therapeutic options in PSCC.

### 3.1. Live Vector-Based Vaccines

There has been a paradigm shift from using generic vaccines toward the generation of targeted vaccines specific for an individual patient’s tumor profile with dendritic cells as carriers for neoantigens to activate T-cells. An additional target for therapeutic vaccination may involve generating a potent CD8+ T-cell response to aggressively recognize and kill HPV-infected tumor cells via live vector-based vaccines. Oncolytic viral-vector vaccines have been explored for the management of HPV-induced malignancies, particularly PSCC [24]. The VSV-GP-based anti-HPV-16 therapeutic vaccines have been shown to preferentially target HPV-16 E2, E6, and E7 antigens [24].

Live-attenuated viral platforms, including lymphocytic choriomeningitis virus-based systems encoding HPV16 E6/E7, have induced potent HPV-specific CD8+ T-cell responses and antitumor activity in preclinical HPV-positive tumor models [18]. Combination with PD-1/PD-L1 blockade has improved activity in some preclinical settings, supporting the broader concept that vaccine-induced T-cell priming may be enhanced by checkpoint inhibition [25,26].

### 3.2. Peptide-Based Vaccines

Peptide-based vaccines deliver defined antigenic peptides to stimulate tumor-specific T-cell responses. Prior studies have highlighted the role of peptide vaccines in prostate, renal cell, and urothelial cancers [19]. These vaccines cause the activation of cytotoxic T lymphocytes (CTL) by presenting short antigenic peptides through major histocompatibility complex (MHC) molecules [27]. Peptide vaccines have practical advantages, including relative safety, manufacturing simplicity, and the ability to combine multiple epitopes. However, their efficacy as monotherapy has often been limited by weak immunogenicity, HLA restriction, inadequate antigen presentation, and tumor-induced immune suppression. Strategies to improve activity include immune adjuvants, toll-like receptor agonists, multi-epitope vaccine design, optimized delivery systems, and combination with immune checkpoint blockade [28]. This is particularly relevant for HPV-positive PSCC, where regulatory T-cells (Tregs) and exhausted T-cell phenotypes have been identified in the tumor microenvironment (TME) and may be responsible for impaired vaccine responses [24].

Clinical trials investigating therapeutic peptide vaccines, including HPV-16 synthetic long peptide (HPV16-SLP) and HPV-16 E6 peptides (PepCan) [29], are ongoing in early phases; however, therapeutic efficacy is strongly dependent upon the HLA genotype of the individual since antigen presentation and immune activation could be influenced by HLA variability [19].

In large or advanced tumors, vaccine monotherapy has generally produced limited durable clinical benefit, whereas combinations with immune checkpoint inhibitors (ICIs), chemotherapy, or radiotherapy may enhance antitumor activity. For PSCC, peptide vaccines may be most relevant in HPV-positive disease and potentially in minimal residual disease or earlier-stage settings, where tumor burden and immune suppression may be lower [30,31].

Because peptide vaccine efficacy is influenced by HLA genotype, HLA-informed or multi-epitope vaccine design will be important for future PSCC studies. Delivery systems such as liposomes, polymeric nanoparticles, or other carrier platforms may further improve antigen stability, Antigen Presenting Cells (APC) uptake, and immune activation [19].

### 3.3. Nucleic Acid Vaccines

DNA- and RNA-based vaccines represent a promising class of therapeutic strategies in HPV-associated malignancies, with potential applicability in PSCC. DNA vaccines encoding HPV E6 and E7 have demonstrated immunogenicity in HPV-associated malignancies. One of the most clinically advanced examples, VGX-3100, has shown the ability to induce HPV-specific T-cell responses and promote lesion regression in patients with high-grade cervical intraepithelial neoplasia (CIN2/3). Although these results provide proof-of-concept for HPV-targeted DNA vaccination, their applicability to invasive PSCC remains to be established [32,33].

Recent clinical evidence has further supported the potential of therapeutic DNA vaccination in HPV-driven malignancies. In a multicenter phase IIa study, Hillemanns et al. evaluated the HPV16-targeted DNA vaccine VB10.16 in combination with atezolizumab in patients with persistent, recurrent, or metastatic HPV16-positive cervical cancer. The combination demonstrated a favorable safety profile, induced HPV16-specific T-cell responses, and showed encouraging antitumor activity, particularly among patients with PD-L1-positive tumors. Although these findings were generated in cervical cancer rather than PSCC, they provide important clinical proof-of-concept that therapeutic DNA vaccines can successfully induce antigen-specific immunity and may enhance the efficacy of immune checkpoint inhibition in HPV-driven malignancies [34]. Additionally, in the phase I/II HARE-40 study, Ottensmeier et al. evaluated BNT113, a lipoplex-formulated uridine mRNA vaccine encoding HPV16 E6 and E7 antigens, in patients with HPV16-positive cancers. The vaccine demonstrated a favorable safety profile and induced HPV-specific cellular immune responses in the majority of evaluable patients, including expansion of E6/E7-reactive CD4+ and CD8+ T-cells. Notably, disease control was observed in patients with advanced HPV16-driven malignancies despite prior treatment exposure, providing important clinical proof-of-concept that therapeutic mRNA vaccination can generate biologically meaningful antitumor immunity. Although PSCC was not included in this study, these findings support continued investigation of mRNA-based vaccine platforms targeting HPV-derived antigens in HPV-positive PSCC [35].

A major limitation of DNA vaccines has been their relatively low immunogenicity. Several strategies have been developed to enhance their efficacy, including co-delivery of immunostimulatory molecules such as FMS-like tyrosine kinase 3 ligand to improve dendritic cell activation and antigen presentation. Additional approaches include fusion of HPV antigens with immune-enhancing molecules such as calreticulin or heat shock protein 70 (HSP70), which promote cross-presentation and strengthen cytotoxic T-cell responses. Preclinical studies have demonstrated that such modifications can significantly improve the immunogenicity of DNA vaccines compared to unmodified constructs [24].

Another mode of improving DNA vaccine immunogenicity is nanotechnology-based delivery systems [33]. Electroporation, for example, enhances cellular uptake of the virus vector DNA, resulting in a greater expression of antigens and immune activation. A trial evaluating electroporation-enhanced administration of VGX-3100 has shown an improvement in both T-cell responses and lesion recovery compared to the traditional intramuscular injection procedure in [36].

In contrast to DNA vaccines, RNA-based vaccines offer several theoretical advantages over DNA platforms, including rapid antigen expression, lack of genomic integration, and greater flexibility in vaccine design. Messenger RNA (mRNA) vaccines have emerged as a highly adaptable platform capable of encoding full-length antigens or multiple epitopes. Once delivered, typically via lipid nanoparticle systems, mRNA is translated into the cytoplasm, enabling efficient antigen production and presentation through both MHC class I and II pathways [36].

mRNA vaccines targeting HPV E6 and E7 have demonstrated strong immunogenicity in preclinical models, inducing robust cytotoxic T-cell responses and IFN-γ production. Their modular design also allows for incorporation of patient-specific neoantigens, making them attractive candidates for personalized immunotherapy approaches in PSCC. More recently, clinical development of HPV-targeted nucleic acid vaccine platforms has continued to demonstrate encouraging immunogenicity and expansion of antigen-specific T-cell responses in HPV-associated malignancies, supporting further evaluation of these approaches in rare HPV-driven tumors such as PSCC [36]. Furthermore, mRNA vaccines may be combined with ICIs or adjuvants to enhance T-cell activation and overcome tumor-induced immune suppression [36].

Despite these advantages, several challenges remain. RNA vaccines are inherently unstable and susceptible to degradation, necessitating advanced delivery systems to ensure efficient in vivo uptake and translation. Additionally, as with DNA vaccines, their clinical efficacy may be limited by tumor-immune evasion mechanisms, including impaired antigen presentation and T-cell exhaustion [36].

### 3.4. Dendritic Cell-Based Vaccines

**Figure 3:**
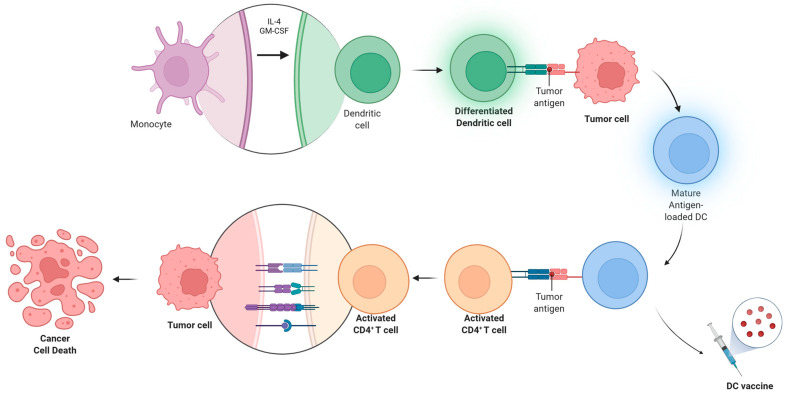
Generation and therapeutic application of dendritic cell vaccines in PSCC. Autologous monocytes are differentiated into dendritic cells ex vivo and subsequently loaded with tumor-associated antigens, HPV-derived antigens, neoantigens, or tumor lysates. Following reinfusion, activated dendritic cells present antigens to CD4+ and CD8+ T-cells, promoting tumor-specific immune responses. In PSCC, this strategy may facilitate recognition of HPV-derived E6/E7 antigens in HPV-positive tumors and patient-specific neoantigens in HPV-negative disease. However, clinical efficacy remains investigational and may require combination with immune checkpoint blockade or other immune-modulating approaches.

Dendritic cell (DC)-based vaccines have been identified as a promising strategy of immunotherapy for patients with a variety of HPV-associated diseases including PSCC. DCs present antigens to CD4+ T helper lymphocytes and CD8+ CTLs and are essential for killing tumors. Immunization with DC leads to the antitumor response of CD4+ helper and CD8+ cytotoxic lymphocytes. Patients undergo DC-based vaccination by first retrieving autologous monocyte-derived DCs, proliferating those cells and dressing them in autologous tumor-associated antigens (TAAs) or tumor cell lysate, and subsequently introducing them back into these patients to destroy the immune response of T-cells. However, certain barriers exist, including limitation of the immune response by the tumors and the need of combinations of vaccines to enhance performance [21] (Figure 3).

DC vaccines have been studied in several malignancies, including prostate cancer, melanoma, renal cell carcinoma, and glioblastoma. They can induce measurable antigen-specific immune responses, although clinical efficacy has often been modest in advanced tumors [30]. Sipuleucel-T, an autologous cellular immunotherapy approved for metastatic castration-resistant prostate cancer, demonstrates the clinical feasibility of this general approach but should not be interpreted as direct evidence of efficacy in PSCC [21,36].

Clinical studies have identified that dendritic cells vaccines are usually hampered by low rate of dendritic cell maturation and insufficient amount of antigen presentation [37,38]. There have been initiatives to counteract these problems through novel strategies such as adjuvant-based DC activation, RNA-loaded DCs, and combination treatment together with ICIs. For example, clinical trials showed that HPV E6 and E7 peptide-pulsed DC vaccines could significantly enhance the infiltration of HPV-specific T-cells within the tumors and increase tumor regression [31]. Additionally, TLR agonists, such as CpG-ODNs and poly I:C, have been used to up-regulate DC activation and antigen presentation and resulted in better antitumor responses in the preclinical models. Such outcomes suggest that better DC maturation strategies and more deliberate antigen delivery strategies offer promise for effective DC-based vaccines for PSCC [39].

Several phase I–III studies have investigated the use of DC vaccines in a broad array of tumors including melanoma, renal cell carcinoma and glioblastoma. However, one major challenge experienced in these studies was the lack of durability of the DC-mediated immune responses. Several tumors developed mechanisms to suppress immune activity through T-cell fatigue and upregulation of PD-L1 on immune cells [37]. As such, it may be necessary to combine DC vaccination with ICIs or cytokine therapy to overcome immune suppression within tumors [37,38].

Immune profiling studies suggest that dendritic cell function may be impaired in some patients with HPV-associated PSCC, including reduced activation markers and weaker lymphoproliferative responses. A recent study investigating anti-cancer immune cells in 30 patients with PSCC, 66.6% of whom were known to be HPV-positive, found that patients with high-risk HPV exhibited significantly lower levels of circulating activated DCs and expression of co-stimulatory molecules (CD80, CD86 and HLA-DR) compared to those in HPV-negative patients and healthy controls [40]. These findings support continued investigation of strategies that improve DC maturation and function, such as toll-like receptor agonists, cytokine support, RNA loading, and combination with immune checkpoint blockade.

## 4. Personalized Vaccine Subtypes

### 4.1. Combinatory Vaccine Therapy with Immune Checkpoint Inhibitors

Recent clinical studies have renewed interest in therapeutic HPV-targeted vaccination through combination immunotherapy approaches. Although promising results have been obtained during preclinical and early-phase clinical trials, none have been globally approved for clinical use due to inconsistency in their clinical efficacy. To date, Several contemporary vaccine platforms, including HPV16 E6/E7-targeted vaccines combined with ICIs, have demonstrated encouraging immunogenicity and antitumor activity in HPV-associated malignancies. Emerging data presented from studies involving HPV-driven cervical and head and neck cancers suggest that vaccine-induced expansion of tumor-specific T-cells may enhance responsiveness to PD-1-directed therapy and improve immune infiltration within the tumor microenvironment [41]. Although these studies were not conducted in PSCC, they provide important proof-of-concept supporting further evaluation of vaccine-based combination strategies in rare HPV-associated tumors [42]. One study indicated that combination approaches, especially ones integrating PD-1 inhibitors and peptide-based vaccines, showed synergistic effects in experimental models [43]. A phase II clinical investigation of the PD-1 inhibitor nivolumab combined with the HPV16 peptide vaccine demonstrated that this combination achieved 33% response rate, a better outcome relative to nivolumab monotherapy or the HPV peptide vaccine alone. ISA101 is now under investigation both with utomilumab (anti-4-1BB) and cemiplimab (an-ti-PD1) [43].

Another study used a mathematical model to assess vaccine–ICI synergy and found that adjuvant use is superior to either treatment alone. The model incorporates some key variables including DC activation, CD4+/CD8+ T-cell proliferation, and cytokine dynamics (IL-12, IL-2). By administering a combination of vaccine with PD-1 inhibitor, higher T-cell infiltrates, and tumor regression were found compared to increasing the dose of either single agent, thus validating their observations for synergy [44].

A key limitation of checkpoint inhibitors in PSCC is that tumors may have a very low density of tumor-infiltrating T-cells (TILs), thus limiting the effectiveness of PD-1/PD-L1 blockade. Cancer vaccines may address this problem by increasing the pool of tumor-specific T-cells to prime the immune system for checkpoint blockade therapy. RNA and peptide-based vaccines targeting for TAAs or neoantigens in PSCC could follow this approach to elicit an immune response before the application of checkpoint inhibitors. The approach has been tested in bladder carcinoma and prostate carcinoma, suggesting that preloading of the immune system with activated T-cells enhances the clinical efficacy of checkpoint blockade [45].

The efficacy of vaccines can be further compromised by several intrinsic and extrinsic factors associated with the tumor, including loss of antigen presentation due to mutations in HLA or β2-microglobulin involved in the recognition of T-cells, wnt/β-catenin signaling upregulation to exclude T-cell infiltration and evade immune recognition, and expansion of immunosuppressive cells within the TME (Tregs, MDSCs, and TAMs) which decrease efficacy of the vaccine [46]. (Table 1) [47]. Another emerging factor that may influence vaccine efficacy is the tumor-associated microbiome. Growing evidence suggests that microbial communities can modulate antigen presentation, dendritic cell activation, and responsiveness to immunotherapy. Although the microbiome of PSCC remains poorly characterized, future studies may help determine whether microbial composition influences vaccine-induced antitumor immunity and clinical outcome [48].

### 4.2. Spatial Transcriptomics and Single Cell-RNA Sequencing in Target Identification

Spatial transcriptomics (ST) and single-cell RNA sequencing can support vaccine development by defining antigen expression, malignant-cell states, immune-cell composition, and suppressive niches within the PSCC tumor microenvironment [47].

The ability of ST to map expression of tumor-specific antigens in different tumor compartments opens a window for personalized cancer vaccine development. Unlike standard vaccine mechanisms that identify a common tumor-associated antigen, ST allows for the identification of patient-specific neoantigens in regions of aggressive tumor expression [49]. This allows for the design of precision vaccines that launch highly specific immune responses based on the individual tumor architecture. ST also reveals important information regarding tumor-immune interactions, including specific directions regarding localization of activation states of TILs [49]. This analysis reveals spatially localized exhausted T-cells within the tumor core and leading edge, which may indicate that these areas are favored for vaccine-mediated immune reinvigoration.

Tumor subpopulations with high metastatic potential such as the MMP3+SPP1+ malignant subset driving EMT and immune resistance, were identified with the use of ST [50]. The TME of these subtypes is associated with upregulated NF-κB and TLR signaling pathways, both of which modulate immune responses promoting tumor survival. These findings imply that therapeutic vaccines should be developed to target such TAAs or modulate those immune-suppressive signaling pathways to arm immune clearance of PSCC cells (see Table 2) [47].

ST data further demonstrates that expression of Casein kinase 2 (CK2a), a key oncogene, is spatially enriched in malignant areas within PSCC. Because CK2a is part of oncogenic signaling and immune evasion, it becomes an important target for this vaccine approach. A vaccine inducing an immune response to tumor cells expressing CK2a may reduce tumor burden and enhance effects of other therapies, including ICIs [47].

Future studies should integrate ST with proteomics, immunopeptidomics, and functional T-cell assays to determine whether these targets can generate clinically meaningful antitumor immunity.

Therapeutic vaccine platforms under investigation in other solid tumors and HPV-associated cancers may be relevant to PSCC, particularly for HPV-positive tumors. However, the absence of PSCC-specific prospective vaccine trials means that these approaches should be presented as translational opportunities rather than established therapeutic options.

## 5. Emerging Trends and Future Directions

### 5.1. AI in Vaccine Development

Artificial intelligence (AI) may play a particularly important role in PSCC because the rarity of the disease limits the availability of large prospective datasets and traditional vaccine-development pipelines. Rather than serving as a general vaccine-design tool, AI may facilitate several specific aspects of personalized vaccine development [50]. First, machine-learning algorithms can prioritize candidate neoantigens identified through whole-exome and RNA sequencing by predicting antigen processing, presentation, and immunogenicity [50]. Second, AI-assisted HLA-binding prediction platforms can improve identification of epitopes most likely to generate effective CD8+ and CD4+ T-cell responses. Third, integrative multi-omics models combining genomic, transcriptomic, spatial transcriptomic, and immunopeptidomic data may enable ranking of clinically relevant vaccine targets while reducing false-positive antigen selection [51]. Fourth, AI-driven biomarker discovery could identify patients most likely to benefit from vaccine-based approaches through characterization of immune-inflamed, immune-excluded, or immune-desert tumor phenotypes [51]. Finally, AI may support trial enrichment strategies by improving patient selection and optimizing the design of biomarker-driven studies in rare malignancies such as PSCC. Although these applications remain investigational, they may accelerate development of individualized vaccine strategies for both HPV-positive and HPV-negative PSCC [51].

### 5.2. Advances in Nanotechnology

Nanotechnology-based delivery systems may improve vaccine efficacy by enhancing antigen stability, promoting uptake by APCs, enabling co-delivery of antigens and adjuvants, and supporting controlled antigen release [52,53,54,55,56,57]. These properties are especially relevant for peptide and RNA vaccines, which may otherwise have limited stability or inefficient delivery.

Lipid nanoparticles are a major delivery platform for RNA-based vaccines because they protect RNA from degradation and facilitate cellular uptake. Polymeric nanoparticles, including PLGA-based systems, can encapsulate antigens and immunostimulatory molecules and may support sustained antigen release. Other platforms, such as liposomes, gold nanoparticles, and hybrid nanoparticles, have been explored for antigen delivery and immune activation in preclinical cancer vaccine models [58].

For PSCC, nanotechnology-enhanced vaccines remain hypothetical but potentially valuable. Improved delivery may help increase dendritic cell uptake, enhance antigen presentation, and generate stronger cytotoxic T-cell responses [59].

### 5.3. Challenges and Current Limitations

Despite favorable preclinical and early clinical outcomes, meaningful clinical benefit from therapeutic vaccines has not yet been demonstrated in PSCC. Several biological and clinical factors likely contribute to this limitation. PSCC exhibits substantial heterogeneity with respect to HPV status, antigen expression, mutational landscape, and immune microenvironment composition, complicating the identification of universally effective vaccine targets. In addition, immune evasion mechanisms, including progressive T-cell exhaustion, impaired dendritic cell activation, reduced antigen presentation, and the presence of immunosuppressive cellular populations within the tumor microenvironment, may limit the durability and magnitude of vaccine-induced immune responses. Variability in HLA-restricted antigen presentation may further influence vaccine efficacy across patients. Collectively, these findings suggest that therapeutic vaccines are unlikely to achieve substantial clinical benefit as monotherapy and may require combination strategies with immune checkpoint inhibitors or other immune-modulating approaches. Continued biomarker validation will be necessary to support scientifically robust patient selection and optimize future vaccine-based clinical trials [22,40]. The low prevalence of PSCC also presents challenges in recruiting patients for vaccine clinical trials due to low enrollment and reduced statistical power. Additionally, identifying optimal target vaccines and methods of delivery remain principal barriers to maximally improving the efficacy of vaccines. Other technical challenges such as low levels of neoantigen load, immune evasion due to PD-L1 overexpression, and lack of durable T-cell memory also undermine vaccine-based options [22,40].

Furthermore, many promising vaccine concepts discussed in this review are supported by evidence from other HPV-associated malignancies or preclinical models rather than PSCC-specific trials. Therefore, claims regarding efficacy in PSCC should remain cautious. Future studies should include correlative immune analyses to determine whether vaccines generate target-specific T-cells, whether those cells infiltrate tumors, and whether immune activation translates into clinical benefit [11,13,60].

### 5.4. Future Directions and Emerging Trends

To overcome current limitations, future research should address functional improvements of vaccine delivery, antigen presentation, and modulation of the TME to further enhance the vaccine efficacy in PSCC. AI prediction of neoantigens and optimization of vaccine design is another future dimension for accelerated development of vaccines [61,62]. Recent advances in nanotechnology can also be harnessed for better delivery of vaccines and their stability, and for the increases in immunogenicity and clinical response rates. Biomarker-based clinical trials will also be needed to optimize individualized treatment in which those patients who are most likely to respond to vaccine-based therapy can benefit from immunotherapeutic intervention [63]. Recent clinical programs evaluating therapeutic HPV-targeted immunotherapy have increasingly adopted biomarker-driven and combination-based approaches, highlighting the importance of patient selection and translational immune monitoring for future vaccine development in HPV-associated malignancies [63]. This will also include improved international collaboration concerning research alongside better recruitment of patients to vaccine trials. Greater interdisciplinary collaboration and coordination of legislation should increase the role of AI in vaccine development strategies to efficiently impart safety and transparency without raising privacy-based ethical issues for patient data. Because most current vaccine concepts are extrapolated from other HPV-associated malignancies, future PSCC-specific translational and clinical studies will be essential to validate these approaches.

## 6. Conclusions

Therapeutic vaccines represent a promising but unproven strategy in PSCC. HPV-positive tumors provide the clearest rationale through expression of E6 and E7 viral oncoproteins, while HPV-negative disease will require more individualized antigen-discovery approaches. Current evidence supports further investigation of vaccines as part of rational combination strategies rather than as stand-alone therapy. Future progress will depend on PSCC-specific translational studies, biomarker-driven clinical trials, improved antigen validation, and integration of emerging technologies such as spatial profiling, AI-assisted antigen prediction, and advanced delivery systems.

## Figures and Tables

**Table 1:** New insights on vaccine development.

New Insight	Relevance to PSCC Vaccines	Potential Applications
Combination Therapy with Oncolytic Viruses	Immune response enhancement potentially improving vaccine efficacy in PSCC.	Potential for personalized cancer treatment strategies.
Tumor-Associated Microbiome	Modulates immune responses to vaccination, Influences antigen presentation and immune checkpoint expression.	Improve immune activation in PSCC patients.
HLA Typing for Personalized Vaccine Strategies	HLA diversity impacts neoantigen presentation	Personalized vaccine development for PSCC patients using HLA typing to predict strong antigen presentation and response likelihood.
Immune Memory Formation in PSCC Vaccination	Long-term immune memory after vaccination is crucial to prevent recurrence	Developing vaccine formulations that promote tissue-resident memory T-cells to sustain long-term antitumor immunity.
Bioinformatics and Multi-Omics Integration	AI-driven bioinformatics can predict optimal vaccine antigens through integration of spatial transcriptomics and multi-omics data	Leveraging computational approaches to refine vaccine design, ensuring selection of highly immunogenic targets in PSCC.

**Table 2:** Therapeutic Vaccine Modalities for solid tumors with relevance to PSCC.

Vaccine Modality	Example Clinical Trials	Target Antigens	PSCC Relevance
Peptide-Based Vaccine	ISA101 Phase II [44] PDS0101 (HPV+ HNSCC) [31]	Tumor-associated antigens Synthetic long peptide HPV16 E6/E7),	High relevance in HPV+ PSCC and shared TAAs
DNA Vaccine	VGX-3100 (HPV-associated lesions) [33]	HPV oncogenes (E6/E7), tumor antigens	HPV16/18 & E6/E7 are relevant in PSCC context
mRNA Vaccine	BNT113 (BioNTech) Phase I (HPV+ OPC) [35]	HPV16 E6/E7	Rapid adaptability Targets HPV oncogenes High safety profile
Dendritic Cell-Based Vaccine	Sipuleucel- (prostate cancer) [36]	Autologous DCs loaded with tumor antigens or RNA	Has shown immunogenicity in squamous tumors Scalable to PSCC
Live Vector-Based Vaccine	VSV-GP-based anti-HPV-16 [23]	HPV oncogenes (E6/E7)	Promising results in cervical and head & neck cancers PSCC patients with HPV positivity may benefit from E6/E7 targeting.

## Data Availability

No new data were created or analyzed in this study. Data sharing is not applicable to this article.

## Abbreviations

The following abbreviations are used in this manuscript:

**Abbreviation**	**Definition**
AI	Artificial intelligence
APC	Antigen-Presenting Cell
CIN2/3	Cervical intraepithelial neoplasia grade 2/3
CK2a	Casein kinase 2 alpha
CpG-ODNs	Cytosine-phosphate-guanine oligodeoxynucleotides
CTL	Cytotoxic T lymphocyte
DC	Dendritic cell
GM-CSF	Granulocyte–macrophage colony-stimulating factor
GVAX	Granulocyte–macrophage colony-stimulating factor-secreting tumor vaccine
HLA	Human leukocyte antigen
HLA-DR	Human leukocyte antigen-DR
HNSCC	Head and neck squamous cell carcinoma
HPV	Human papillomavirus
HSP70	Heat shock protein 70
ICIs	Immune checkpoint inhibitors
IFN-γ	Interferon gamma
IgG2	Immunoglobulin G2
IL-2	Interleukin-2
IL-4	Interleukin-4
IL-12	Interleukin-12
*L. tarentolae*	*Leishmania tarentolae*
MDSCs	Myeloid-derived suppressor cells
MHC	Major histocompatibility complex
MMP3	Matrix metalloproteinase 3
mRNA	Messenger RNA
NF-κB	Nuclear factor kappa B
OPC	Oropharyngeal cancer
p16INK4a	Cyclin-dependent kinase inhibitor 2A/p16INK4a
PSCC	Penile squamous cell carcinoma
Rb	Retinoblastoma protein
SLP	Synthetic long peptide
SPP1	Secreted phosphoprotein 1
ST	Spatial transcriptomics
TAAs	Tumor-associated antigens
TAMs	Tumor-associated macrophages
TILs	Tumor-infiltrating lymphocytes
TLR	Toll-like receptor
TME	Tumor microenvironment
Tregs	Regulatory T-cells
VSV-GP	Vesicular stomatitis virus glycoprotein platform
Wnt/β-catenin	Wingless/integrated beta-catenin signaling pathway
